# Deciphering nanoconfinement effects on molecular orientation and reaction intermediate by single molecule imaging

**DOI:** 10.1038/s41467-019-12799-x

**Published:** 2019-10-23

**Authors:** Bin Dong, Yuchen Pei, Nourhan Mansour, Xuemei Lu, Kai Yang, Wenyu Huang, Ning Fang

**Affiliations:** 1 Department of Chemistry, Georgia State University, Atlanta, GA 30303 USA; 2 Department of Chemistry, Iowa State University, and Ames Laboratory, U.S. Department of Energy, Ames, IA 50011 USA; 3 Center for Soft Condensed Matter Physics and Interdisciplinary Research & School of Physical Science and Technology, Soochow University, 215006 Suzhou, P. R. China

**Keywords:** Heterogeneous catalysis, Fluorescence spectroscopy, Reaction kinetics and dynamics

## Abstract

Nanoconfinement could dramatically change molecular transport and reaction kinetics in heterogeneous catalysis. Here we specifically design a core-shell nanocatalyst with aligned linear nanopores for single-molecule studies of the nanoconfinement effects. The quantitative single-molecule measurements reveal unusual lower adsorption strength and higher catalytic activity on the confined metal reaction centres within the nanoporous structure. More surprisingly, the nanoconfinement effects on enhanced catalytic activity are larger for catalysts with longer and narrower nanopores. Experimental evidences, including molecular orientation, activation energy, and intermediate reactive species, have been gathered to provide a molecular level explanation on how the nanoconfinement effects enhance the catalyst activity, which is essential for the rational design of highly-efficient catalysts.

## Abstract (web summary)

Nanoconfinement effects on changing molecular transport and reaction kinetics in heterogeneous catalysis have been widely recognized. Here, the authors design a core-shell nanocatalyst with aligned linear nanopores to uncover nanoconfinement effects on catalytic activity and adsorption strength by single molecule imaging.

## Introduction

Confining catalytic active centres in well-defined space is a rational strategy that we have learnt from nature’s catalysts, enzymes, for designing effective catalysts. Nanoporous materials, such as zeolites, metal-organic frameworks (MOFs), and covalent-organic frameworks (COFs), have contributed to improving the performance of many catalytic systems by assisting in the chemical bond formation/breaking^1–4^, altering product selectivity^5–7^, and/or optimising mass transport^8–10^ and adsorption–desorption equilibrium^11,12^. Despite intensive research efforts with either theoretical calculations in simplified systems^13–16^ or experimental measurements at the ensemble level^17–21^, the quantitative understanding of the nanoconfinement effects in catalysis at the molecular level remains largely unexplored experimentally.

Single molecule localisation-based super-resolution microscopy^22,23^ has emerged in the past decade to offer nanoscale spatial resolution for visualising heterogeneous activities on single nanocatalysts, such as layered double hydroxides^24^, zeolites^25–29^, metal nanoparticles^30–33^, and semiconductors^34–37^. Heterogeneous features of local microenvironment inside 2D mesoporous materials, such as the defects and blocks, have also been uncovered in optical imaging experiments^38–41^.

Here we report strikingly unusual behaviours of catalytic reaction dynamics under variable nanopore morphologies. With the experimental evidence on a wide range of catalytic parameters, including molecular adsorption and diffusion, catalytic reaction kinetics, molecular orientation, activation energy, and intermediate reaction species, our explanation of the puzzling observations leads to the discovery of the nature of the nanoconfinement effects in heterogeneous catalysis at the molecular level.

## Results

### Single-molecule imaging on core–shell nanoporous catalysts

**Fig. 1:**
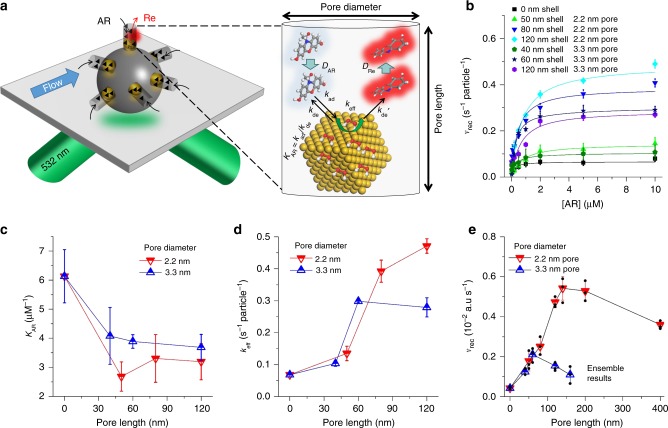
Reaction kinetics in confining materials with variable nanopore morphologies. **a** Schematic of single-particle single-molecule imaging setup (left) and chemical conversion processes inside nanopore (right). **b** Reaction kinetics for different nanopore lengths and diameters at single particle single molecule level. Adsorption/desorption equilibrium constant *K*_AR_ **c** and rate constant *k*_eff_ **d** are obtained from fitting the single particle single molecule kinetics data with the DLH model. **e** Ensemble results of reaction rates of nanocatalysts with variable porous shell thickness and pore diameter are shown at same amount of particle concentrations (~10^12^ particles mL^−1^). It should be noted that the shallow excitation depth of TIRFM (<250 nm) makes it challenging to compare single particle catalytic activities of thicker shells (>150 nm) to those with thin shells (40–120 nm) quantitatively. The error bars are calculated uncertainties from fitting catalytic reaction kinetic data over 40 single nanocatalysts for each nanopore morphology (**b** and **c**) and calculated standard deviation (s.d.) from three trials of ensemble measurements (**e**), respectively

The single molecule imaging experiments were conducted on a model nanocatalyst of well-defined multilayer structure, which is composed of an optically transparent solid silica dioxide (SiO_2_) core, a porous silica shell with aligned linear pore structure (mSiO_2_), and platinum nanoparticles (Pt NPs) sandwiched between the core and shell (Supplementary Fig. 1). The synthesis and characterisation of the nanocatalysts are described in detail in the Supplementary Methods, Supplementary Tables 1 and 2, and Supplementary Figs. 1–7. A fluorogenic reaction system was used to study the activity of the core–shell nanocatalysts, where a non-fluorescent reactant molecule (amplex red, AR) is converted to a highly fluorescent product molecule (resorufin, Re) inside the nanopores (Fig. 1a). The fluorescence signal of Re was induced by a circularly polarised (unless otherwise mentioned) green laser (532 nm), collected by a high numerical aperture (NA = 1.2) water-immersion objective, and imaged on a highly sensitive electron multiplying charge coupled device (EMCCD) camera (Supplementary Fig. 8). The nanocatalysts were dispersed in ultrapure methanol, drop-casted, and dried on quartz slides at low concentrations to facilitate single particle studies (Supplementary Fig. 9).

The chemical reaction kinetics of single nanocatalysts with different nanopore morphologies were quantitatively measured at the single-particle, single-molecule level with turn-over resolution (Fig. 1b, Supplementary Figs. 10 and 11). Instinctively, one would expect a decreasing activity when catalytic centre (Pt) is shielded away from reactant molecules by the nanoporous shell. One can also predict such a trend of activity reduction would be more significant for catalysts of thicker nanoporous shell or narrower pore diameter. Surprisingly, the observed experimental results here tell a different story. As shown in Fig. 1b, higher catalytic reaction activity was found counter-intuitively when the nanoporous shell thickness increases from 0 (no shell) to 120 nm and when the nanopore diameter decreases from 3.3 to 2.2 nm. Heterogeneous catalysis is often an interplay between mass transport and chemical conversion, especially when catalytic centres are inside confining materials^42,43^. Therefore, a diffusion-coupled Langmuir–Hinshelwood (DLH) kinetic model^43^ was used for fitting the reaction kinetics data in Fig. 1b, giving adsorption–desorption equilibrium constant *K*_AR_ (Fig. 1c) and apparent reaction rate constant *k*_eff_ (Fig. 1d). Catalytic activities of these nanocatalysts were also measured using ensemble experiments (Fig. 1e, Supplementary Fig. 12) and the results agreed with the single-molecule data.

### Molecular orientation in nanopores

**Fig. 2:**
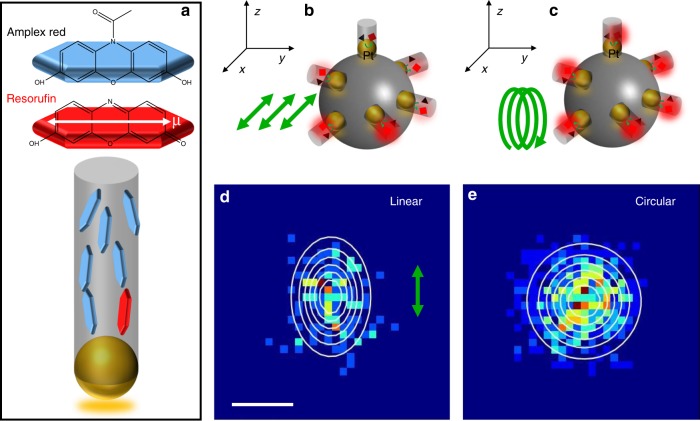
Molecular orientations in nanopore using single molecule fluorescence polarisation microscopy. **a** Dipole moments of AR, Re molecules and schematic view of molecular arrangement inside nanopore. Single-particle single-molecule imaging experiments under linearly polarised excitation (s-pol, **b**, **d**) and circularly polarised excitation (c-pol, **c**, **e**). Scale bar equals to 200 nm

In heterogeneous catalysis, the chemical conversion rate over the molecular adsorption energy follows a volcano shape curve^44^. High reaction rates are accomplished by an optimal adsorption strength of reactive species on catalytic centres^45^. Adsorption of a molecule in a 3D enclosed space with dimensions comparable to the size of the molecule can be very different from that on a freely accessible surface. From the single molecule imaging experiments, the adsorption strength (*K*_AR_) of AR molecules on Pt NPs inside the nanopores of different lengths was measured to be ~2 times smaller than that without the nanoporous shell (Fig. 1c). The aromatic molecular structure of AR and Re suggests that the strongest adsorption occurs when it lays down flat on the top surface of Pt NPs by forming pi-bonds with the metal surface^46–50^. However, we cannot rule out the possibility that the complex surface structure of Pt NPs can contribute to the heterogeneity in adsorption behaviour of AR on Pt surface. Nonetheless, we hypothesise that the decreased molecular adsorption strength inside the nanopore is caused by AR molecules approaching to and adsorbing on Pt NPs at standing orientations (Fig. 2a), where the molecular long axis aligns perpendicular to Pt NPs surface and parallel with the long axis of the nanopore in our simplified model.

Single molecule fluorescence polarisation microscopy imaging^51,52^ was used to verify this hypothesis (Supplementary Fig. 13). The Re molecule has absorption and emission dipole moments (*µ*) along the long axis of their planar structure^53^ (Fig. 2a). The generated Re molecules were localised (Supplementary Fig. 14) for reconstructing super-resolution images of their distributions on individual nanocatalysts. An elliptical distribution of Re molecules was observed under the linearly polarised excitation (s-pol, Fig. 2b, d) with more molecules found along the polarisation direction. Only Re molecules, whose absorption dipole moments were aligned with the excitation light polarisation direction, generated sufficient photons for detection. However, when switching to a circularly polarised (c-pol) excitation light, this asymmetrical distribution pattern disappeared (Fig. 2c, e) and more Re molecules were detected (Supplementary Table 3), because the circularly polarised beam could equally excite all Re molecules with different absorption dipole moment orientations. These results unambiguously suggest that the long axis of Re molecules is aligned with the linear nanopores. Such confinement effects on molecular orientations inside nanometer-size pore have also been reported previously^54–58^. Due to technical limitations, it is impossible to directly monitor the adsorption and orientation of the non-fluorescent reactant (AR) in a confined nanopore. However the molecular structure of AR is similar to Re, the preferential orientation of Re molecules in nanopores could be applied to the case of AR molecules. Therefore, the hypothesis of reduced adsorption strength of AR caused by it approaching and adsorbing on Pt NPs with standing orientations is reasonable.

### Dependence of activation energy on pore diameter and length

Unlike molecular adsorption strength which is dependent on nanopore diameter but insensitive to nanopore length (Fig. 1c), the catalytic activity in nanopore depends upon both pore diameter and length (Fig. 1b, d): the kinetic rate constant increases by up to seven times as the shell thickness increases from 0 to 120 nm. The standing orientation of AR in nanopores explains not only the decreased molecular adsorption strength, but also the faster chemical conversion rate. The catalytic reaction of AR on Pt NPs follows a two-step single electron transfer mechanism^59,60^ (Supplementary Figs. 15 and 16). In the initial step of the reaction, phenol group on AR transfers one electron to reactive oxygen species (ROS), e.g., chemisorbed oxygen on Pt surface^61^, to produce the intermediate species of AR radical (AR^●^). The standing AR molecules in nanopores would increase the probability of the phenol group reacting with ROS as it directly faces toward the surface of Pt NPs, therefore increasing the formation rate of AR^●^.

The increased kinetic rate constant was consistent with the measured activation energy (*E*_a_), which was found to be reduced by a factor of ~1.5 in the presence of nanoporous shell; however, this reduction was independent of pore length for 2.2 and 3.3 nm pore (Supplementary Figs. 17 and 18). The independence of *E*_a_ on nanopore length is expected in theory, as the activation energy is only influenced by the factors related to the chemical nature of the reaction, including temperature, surface ligand, pH, ionic strength, surface electric properties, molecular orientation. All these factors are not variables of nanopore length. Therefore, the reduced *E*_a_ under the nanoconfinement effects still does not explain the dependence of the activity enhancement on nanopore length.

### Enrichment of reaction intermediates in nanopores

We believe that the local concentration of intermediate species (AR^●^) in nanopore is the final piece of puzzle to fully account for the nanoconfinement effects in enhancing the catalytic activities. The disproportionation reaction of AR requires the participation of two AR^●^ to form one AR molecule and AR^+^ cation (Supplementary Fig. 16). AR^+^ can be further converted to the final product Re through hydrolysis. With no nanoporous shell, the intermediate AR^●^ could dissociate from Pt NPs and diffuse into the bulk solution before the combination of two AR^●^ to form Re. With the nanoporous shell, AR^●^ would be temporarily trapped in nanometre-sized confined space once dissociated from Pt NPs, generating high local concentrations of AR^●^ and increasing the formation rate of Re product. Quantitative measurement of molecular diffusion in nanopores using single molecule tracking experiments (Supplementary Figs. 19 and 20) determined the apparent diffusion coefficients *D*_app_ of 0.014 ± 0.008 µm^2^ s^−1^ (2.2 nm pore) and 0.020 ± 0.006 µm^2^ s^−1^ (3.3 nm pore). The much slower mass transport in nanopores comparing to that in bulk condition, *D*_bulk_ = 480 µm^2^ s^−1^, also suggests that enrichment of AR^●^ could be more readily accomplished inside nanopore.

**Fig. 3:**
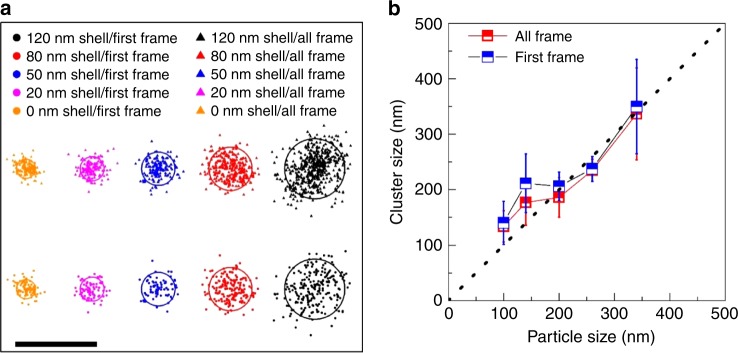
Cluster analysis of Re positions with and without nanoporous shell. **a** Typical cluster distributions of molecular positions of Re when first detected and during their whole life time (all frame) inside nanopore at different pore lengths. The solid line represents the overall average diameter of core–shell nanoparticles. **b** Cluster sizes (mean ± s.d.) from over 20 core–shell nanoparticles versus the overall diameter of core–shell nanoparticles. Scale bar equals to 500 nm

To experimentally verify the proposed nanoconfinement effect of increasing local concentration of intermediate species AR^●^ in nanopores, we used super-resolution imaging to map the locations, where Re molecules were generated in nanoporous materials of variable pore lengths. If AR^●^ species were indeed trapped inside nanopore after dissociation from Pt NPs surface, the production of Re molecules should occur anywhere within the full length of the nanopores, instead of only on/near the surface of the core silica sphere (100 nm in diameter). Super-resolution images of molecular positions (Fig. 3a) at the beginning (down panel, symbol: ●) and through the whole resident time (up panel, symbol: ▲) of Re showed similar cluster sizes, and the cluster sizes of both cases increased with nanopore length (Fig. 3b). These mapping results suggest that Re product molecules were indeed generated at different locations along the full length of nanopores, therefore supporting the concept of trapping AR^●^ inside nanopores. Furthermore, as another strong evidence, the confinement-induced enhancement of catalytic activity would be cancelled out and eventually dominated by the mass transport of reactant molecules as the pore length becomes longer and diffusion is severely restricted as shown from ensemble measurements (Fig.1e). Finally, the enhancement factor of catalytic activity induced by nanoconfinement effect was less pronounced with wider nanopores (e.g. 7.4 ± 0.9 and 4.3 ± 0.6 for 120 nm shell nanocatalysts with 2.2 and 3.3 nm pore, respectively). This reduced enhancement factor is likely a result of higher molecular adsorption strength (Fig. 1c) and faster mass transport rate (i.e., lower local concentrations of AR^●^) in the 3.3 nm pores (Supplementary Fig. 20). Furthermore, this reduced enhancement factor for wider pore also leads to a decrease in the reaction rate at a shorter pore length (Fig. 1e).

In summary, the effects of nanopore morphology, including length (0–400 nm) and diameter (2.2 and 3.3 nm), on the catalytic oxidation of AR were investigated using the single molecule imaging approach. Unusual catalytic activities, namely, lower adsorption strength (*K*_AR_) but higher catalytic activity (*k*_eff_) in the presence of nanoporous shell, were uncovered on the core–shell nanocatalysts. Our efforts to understand these puzzling measurements have led to a quantitative explanation of the nanoconfinement effects on chemical conversion in nanoporous materials at the single molecule level. A full range of experimental evidence, include nanopore confinement-derived molecular orientation, decreased activation energy, and increased concentration of intermediate reactive species inside nanopore, has led to a fundamental understanding of the confinement effect. This molecular-level understanding of the confinement effect leads to the rational design of more efficient catalysts. For example, catalytic reactions that require high local concentrations of intermediates could be optimised with confining reactive centres in nanoscale space where molecular adsorption strength, mass transport, and reaction activation energy could be fine-tuned to form desire products by changing physical features and surface chemistry of the nanoscale space. These designing principals share the same ground as enzymes.

## Methods

### Multilayer nanocatalyst synthesis

The nanocatalysts were prepared by layer-by-layer assembly. The silica cores were synthesised by a seeded growth method, and amino groups were functionalized on the surface of silica cores. After gentle annealing of amino-functionalized silica cores, as-prepared Pt NPs were loaded on the amino-functionalized silica cores. The outer mesoporous silica shells were coated via the hydrolysis of tetraethyl orthosilicate (TEOS) in an ammonia–ethanol–water solution, where hexadecyltrimethylammonium bromide was used as a pore-directing agent. The shell thickness of mesoporous silica shells can be tuned by majorly adjusting the amount of added TEOS. The pore size of mesoporous silica shells can be changed by adding a hexane layer during the mesoporous silica-coating process. Pt NPs were synthesised by reducing potassium tetrachloroplatinate in ethylene glycol with polyvinylpyrrolidone (molecular weight: 40,000). The detailed synthesis procedures of nanocatalysts are included in the Supplementary Methods.

### Single molecule single particle imaging

To achieve best signal to noise ratio of single molecule imaging, the single particle catalysis experiment was carried on a prism-type total internal reflection fluorescence microscope (Supplementary Fig. 8) with setup configurations including an adjustable 100 mW/532 nm continuous wave laser (Oxxius), ×60 water immersion objective (Olympus; numerical aperture: 1.2), iXonEM^+^ Ultra 888 camera (Andor Technology; 1024 × 1024 imaging array; 13 μm × 13 μm pixel size) and fluorescence filter set composed of a 532 nm notch filter (Chroma) and 607/70 bandpass filter (Semrock). The activities of single nanocatalysts were measured by using a fluorogenic oxidation reaction of non-fluorescent AR (10-acetyl-3,7-dihydroxyphenoxazine) to produce highly fluorescent Re (maximum excitation wavelength, *λ*_ex_ = 563 nm and maximum emission wavelength, *λ*_em_ = 587 nm at pH 7.5). Nanocatalysts were deposited on quartz slides using drop-casting method with low particle densities for single particle catalysis. A micro-flow chamber was then assembled with #1.5 coverslip using double-side tape. A reaction solution composed of AR, H_2_O_2_, and phosphate buffer (pH 7.4) was introduced into the micro-flow chamber using a syringe pump (Harvard) with a flow rate set to 20 µl min^−1^. The setup of the single molecule single particle imaging experiments is described in more detail in the Supplementary Methods.

## Supplementary information

Supplementary Information

## Data Availability

All data are available from the corresponding author upon reasonable request.
