# Supplementary material for: Deciphering nanoconfinement effects on molecular orientation and reaction intermediate by single molecule imaging
Source: Nat Commun. 2019 Oct 23;10:4815. doi: 10.1038/s41467-019-12799-x (PMC6811571; doi:10.1038/s41467-019-12799-x)
Supplement: Supplementary file 1 — Supplementary Information [file 41467_2019_12799_MOESM1_ESM.pdf]

Supplementary Information

Deciphering Nanoconfinement Effects on Molecular Orientation and Reaction Intermediate by Single Molecule Imaging

Bin Dong[1], Yuchen Pei[2], Nourhan Mansour[1], Meixue Lu[3], Kai Yang[3], Wenyu Huang[2]*, Ning Fang[1]*

1. Department of Chemistry, Georgia State University, Atlanta, Georgia 30303, United States
2. Department of Chemistry, Iowa State University, and Ames Laboratory, U.S. Department of Energy, Ames, Iowa 50011, United States
3. Center for Soft Condensed Matter Physics and Interdisciplinary Research and College of Physics, Optoelectronics and Energy, Soochow University, Suzhou, 215006, P. R. China

*To whom correspondence should be addressed. E-mail: nfang@gsu.edu; whuang@iatate.edu.

Table of Contents

Supplementary Methods **3**

Preparation of multilayer nanocatalysts 3

Optical setup for single molecule study on single nanocatalysts 6

Single molecule fluorescence polarisation microscopy imaging 8

Ensemble measurement of activation energy of catalytic reaction on nanocatalysts 9

Supplementary Tables **10**

Supplementary Figures **14**

Supplementary Note 1: single molecule imaging of catalytic reaction on single nanocatalysts 21

Supplementary Note 2: density of particles on quartz slide surface 22

Supplementary Note 3: catalytic reaction kinetics at single particle level with turnover resolution .. 23

Supplementary Note 4: catalytic reaction kinetics of 20 nm shell core-shell nanocatalysts 24

Supplementary Note 5: single molecule fluorescence polarisation microscopy 26

Supplementary Note 6: localise single-molecule catalytic events with nanometre precision 28

Supplementary Note 7: molecular mechanism of the oxidation reaction of amplex red to resorufin 30

Supplementary Note 8: chemical reaction activation energy measurement 32

Supplementary Note 9: mass transport of resorufin inside nanopore 34

Supplementary Note 10: the effects of nanopore morphology on mass transport 36

Supplementary References **38**

Supplementary Methods

Preparation of multilayer nanocatalysts

Preparation of 100 nm SiO_2 spheres by seeded growth method [1]. (1) 24 nm SiO_2 seeds: 0.9 mL cyclohexane was added to the mixture of 18.2 mg L-arginine and 13.9 mL ultrapure water to form a two-layer solution. 1.10 mL tetraethyl orthosilicate (TEOS) was added to the cyclohexane layer, after maintaining the above solution at 60.0 ± 0.2 °C for 30 min. The reaction was further kept for 20 hrs at 60 °C. The bottom layer was stored in the refrigerator (Sample A). (2) 45 nm SiO_2 seeds: 4 mL of the 24 nm seeds (Sample A) was diluted with 14.4 mL ultrapure water, and 2 mL cyclohexane was added to form a two-layer solution. After the solution was stabilized at 60 °C for 30 min at 300 rpm, 1.408 mL TEOS was added to the cyclohexane layer, and the mixture was maintained at 60 °C for 30 hrs. The bottom layer was stored in a refrigerator (Sample B). (3) 100 nm SiO_2 spheres (Stöber method): 1 mL of the above 45 nm seeds (Sample B) was mixed with 2.6 mL deionized water, 18 mL ethanol, and 1.7 mL $NH_3 \cdot H_2O$ (~28%). The solution was stabilized at 500 rpm for 1 hr at room temperature. 0.5 mL TEOS was added to the solution dropwise every 30 min for three times (a total of 1.5 mL TEOS). The above solution was maintained at room temperature for 6 hrs under stirring.

Preparation of 5 nm Pt NPs by ethylene glycol reduction [2,3]. 41.5 mg K_2PtCl_4, 505 mg tetradecyltrimethylammonium bromide ($C_{14}TAB$), and 222 mg polyvinylpyrrolidone (PVP-K30, Mw=40,000) were added into 20 mL ethylene glycol (EG). After exchanging the atmosphere with argon, the solution was heated to 140 °C for 2 hrs. As prepared Pt NPs were precipitated by adding 9 times acetone, as to the volume of EG. The precipitate was further washed by an ethanol/hexane mixture (1/4 v/v) for 5 times and stored in 20 mL ethanol.

Preparation of 100 nm SiO_2@5 nm Pt@mSiO_2 nanocatalysts [3-5]. (1) 1 g bare SiO_2 spheres were dispersed in 200 mL isopropanol and 200 μL (3-aminopropyl)triethoxysilane (APTS) were added. After heating and maintained to 80 °C for 2 hrs, NH_2 functionalized SiO_2 spheres were obtained. Obtained NH_2 functionalized SiO_2 spheres were dried in vacuum and annealed at 80 °C in air for 6 hrs, and used directly for the following steps. (2) 0.4 g annealed NH_2 functionalized SiO_2 spheres were dispersed in 120 mL ethanol. The calculated amount of 5 nm Pt NPs in 220 mL ethanol (ca. 2.5 wt.% loading) was added dropwise to the NH_2 functionalized SiO_2 spheres solution with high speed stirring. The resulting 100 nm SiO_2@5 nm Pt solution was separated by centrifugation and washed with ethanol for 5 times. (3) 25 mg (to the mass of pure SiO_2 cores) 100 nm SiO_2@5 nm Pt spheres were dispersed in 10 mL ethanol. After ultrasonication for 30 min, hexadecyltrimethylammonium bromide (C_{16}TAB), 50 mL H_2O and 16.3 mL ethanol were then added to the above solution and ultrasonicated for another 30 min. After 550 μL $NH_3 \cdot H_2O$ (~28%) were then added the above solution and the solution was stirred for 30 min for complete mixing. The calculated amount of TEOS was premixed with 5 mL ethanol and added dropwise into the solution by 3-4 times per 30 min. The solution was stirred for 6 hrs at room temperature. 100 nm SiO_2@5 nm Pt@ mSiO_2 spheres were washed with ethanol for 3 times and redispersed in a mixture of 15 mL methanol and 1 mL concentrated HCl. The mixture was refluxed at 80 °C for 24 hrs to remove surfactants. After refluxing, 100 nm SiO_2@5 nm Pt@mSiO_2 spheres were washed thoroughly with ethanol for 6 times. Supplementary Table 1 summarized the amount of C_{16}TAB and TEOS to obtain nanocatalysts with various shell thickness.

Preparation of 100 nm SiO_2@5 nm Pt@mSiO_2 nanocatalysts with large pore sizes. Pore enlarged 100 nm SiO_2@5 nm Pt@mSiO_2 nanocatalysts were synthesised and modified by a two-layer method as reported in the literature [6]. In the typical synthesis of 100 nm SiO_2@5 nm Pt@mSiO_2 nanocatalysts, 10 mL n-hexane was added to the mixture after the step of the addition of $NH_3 \cdot H_2O$ to form a two-layer solution. The calculated amount of TEOS was then added slowly to the top n-hexane layer. The stirring speed was maintained at 170 rpm after the addition of n-hexane till the completion of the reaction. The reaction time was increased to 12 hrs instead of the aforementioned 6 hrs.

Optical setup for single molecule study on single nanocatalysts

Single-molecule and single-particle imaging experiments were carried out on a prism-based total internal reflection fluorescence (TIRF) microscope (Supplementary Fig. 8a). An adjustable 100-mW 532-nm CW laser (Oxxius, Lannion, France) was focused on the interface between the aqueous sample and quartz slide by a focusing lens, generating a focal spot size of 100 µm × 80 µm. The linearly polarised laser beam was switched to a circularly polarised laser profile by inserting a quarter wave plate (WPMQ05M-532, Thorlabs, Newton, NJ) before the sample. The fluorescence signal was collected by a 60× water immersion objective (Olympus, N.A. = 1.2) and focused onto an Andor iXonEM^{+} Ultra 888 camera (Belfast, Northern Ireland: 1024 ×1024 imaging array, 13 µm × 13 µm pixel size). A fluorescence filter set composed of a 532-nm nortch filter and a 607/70 bandpass filter (Semrock, Rochester, NY) was used to reject scattering signal from the background.

The incident angle of the laser beam at the interface was determined by the angle of the last mirror, which was controlled by a rotational mount (Thorlabs) and a linear translation stage (Thorlabs). The optimal illumination conditions were achieved when the laser spot overlapped perfectly with the view field of the objective by scanning the vertical position of the final mirror. The imaging conditions were fully optimized to achieve maximum illumination depth while maintaining a high signal-to-noise ratio (SNR) for every sample.

To measure the catalytic activities of nanoporous catalysts, a fluorogenic oxidation reaction of non-fluorescent amplex red (10-acetyl-3,7-dihydroxyphenoxazine) to produce highly fluorescent resorufin (λ_{ex} = 563 nm; λ_{em} = 587 nm, at pH 7.5) (Supplementary Fig. 8b) was used. Nanocatalysts were deposited on quartz slides using drop-casting method with low particle densities for single particle catalysis. The density of the nanocatalysts immobilized on the quartz slide was controlled

at low density for single particle catalysis (Supplementary Fig. 9). The sample slide was then assembled to a flow chamber of 40 mm × 6 mm × 0.12 mm with a #1.5 coverslip (Supplementary Fig. 8a). During the imaging experiments, a steady stream of mixture (0.02-10 μM amplex red, 20 mM H_2O_2, and 10 mM pH 7.5 phosphate buffer) was introduced into the flow chamber by using a syringe pump for continuously supplying a constant concentration of reactants over nanocatalysts. The flow rate was set to 20 μL min^{-1}. Highly fluorescent resorufin product molecules were formed at one of many possible reactive Pt NPs on a single nanocatalyst and recorded at an imaging frame rate of 33 fps.

Single molecule fluorescence polarisation microscopy imaging

Linearly polarised laser sources were first used. The polarisation direction of light before illuminating the sample was modulated by a zero-order half-wave plate (Thorlabs). The imaging experiments were first conducted with the s-polarised light (parallel to the interface but perpendicular to the incident light forward direction) for over one hour to collect enough catalytic events on single nanocatalysts. The polarisation of light source was then transformed into the circular polarisation. Single molecule imaging experiments were conducted again on the same nanocatalysts for over an hour. Fluorescence signals from single resorufin molecules were imaged and their positions were located by fitting the intensity distributions. 2D maps of the distribution of resorufin molecules were constructed by their localised positions. The distribution patterns of Re molecules on single nanocatalysts were then analysed and used to explain the restricted motions of AR molecules inside nanopore.

Ensemble measurement of activation energy of catalytic reaction on nanocatalysts

To further study the nanoconfinement effect on the catalytic activity of the core-shell nanocatalysts, temperature control ensemble experiments were conducted using Jasco J-1500 spectrophotometre. The mixture of amplex red, core-shell nanocatalysts and H_2O_2 in 10 mM PBS buffer were kept stirring at 800 rpm during the whole measurement. Once inserting the cuvette into the holder, the fluorescence emission spectra of resorufin were taken at 2-min intervals for 20 min at each temperature. Reaction rates were determined as the slope of the producing resorufin molecules. A calibration curve of a series of known concentrations of resorufin molecules was used to calculate the molar concentration production rate of resorufin. The production rate of resorufin equals the consumption rate of amplex red.

Reaction rates at five temperatures (0, 10, 20, 30, and 40 ^{o}C) were measured. The activation energy (E_a) of the chemical conversion of amplex red to resorufin on platinum nanoparticles can be determined as the slope of log k vs. 1/T based on the Arrhenius equation $k = A\exp^{(-\frac{E_a}{R\mathrm{T}})}$ where k is the rate constant, T is the absolute temperature in kelvin, R (8.314 J mol^{-1} s^{-1}) is universal gas constant, and A is a constant for each chemical reaction. According to the collision theory, A is the frequency of collisions in the correct orientation. The role of nanoporous shell thickness (0 – 120 nm) and nanopore size (2.2 and 3.3 nm) in tuning the activation energies of the chemical conversion on platinum nanoparticles in nanopore were studied.

Supplementary Tables

Supplementary Table 1. The amounts of $C_{16}TAB$ and TEOS for preparing 5 nm Pt NPs loaded nanocatalysts with different $mSiO_2$ shell thickness.

**Thickness of shells (nm)**	20	50	80	120	140	200	400
**$C_{16}TAB$ (mg)**	150	150	160	165	278	515	1200
**TEOS (μL)**	50	200	410	900	1550	3050	7450
**Thickness of shells (nm)**		40*	60*	120*	160*		
**$C_{16}TAB$ (mg)**		150	160	170	280		
**TEOS (μL)**		356	720	1800	2760		

* wider pore samples

Supplementary Table 2. The summary of porous structures for 5 nm Pt NPs loaded nanocatalysts.[a]

**Thickness of $mSiO_2$ shells (nm)**	20	50	80	120	140	200	400	40*	60*	120*	160*
**BET surface area (m^2/g)**	580	770	820	1080	1060	1160	1431	499	770	860	996
**Mesoporosity-BJH des. (cm^3/g)**	0.57	0.91	0.56	0.85	0.62	0.85	1.01	0.58	0.83	1.0	1.0
**Mesoporosity-BJH ads. (cm^3/g)**	0.59	1.19	0.34	0.97	0.69	0.93	0.76	0.78	0.97	1.1	1.1
**Pore size-BJH des. (nm)**	2.4	2.5	2.4	2.3	2.3	2.3	2.0	5.5	3.9	3.4	3.0
**Pore size-BJH**	2.2	2.5	2.3	2.3	2.2	2.2	1.9	3.7	3.7	3.3	3.0

**ads. (nm)**	

a. Mesoporosity was determined by BJH method. Pore size was determined by BJH method using the thickness curve of Harkins and Jura with standard correction. Ads. and des. denote adsorption and desorption. * wider pore samples.

Supplementary Table 3. Comparison of molecular position distribution under linearly polarised and circularly polarised excitation light with 120 nm shell nanocatalysts.

	linear polarisation		circular polarisation	
**Ellipticity**	0.66		0.98	
**Number of molecules**	126		546	
**1/e^2 width**	261 nm	395 nm	377 nm	382 nm
**FWHM**	154 nm	232 nm	222 nm	225 nm

Supplementary Figures

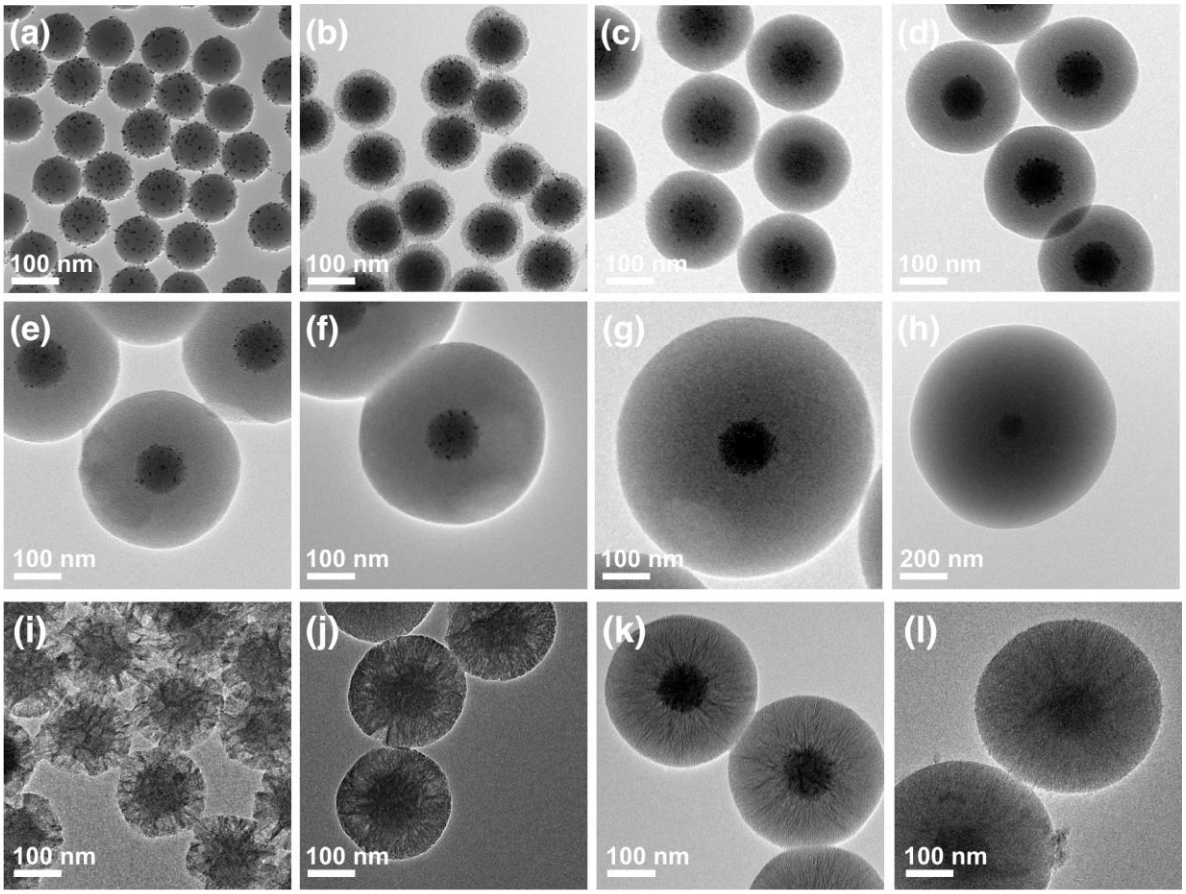

Supplementary Figure 1. TEM images of nanocatalysts. Representative TEM images of nanocatalysts with regular pore (a) no shell; (b) 20 nm shell; (c) 50 nm shell; (d) 80 nm shell; (e) 120 nm shell; (f) 140 nm shell; (g) 200 nm shell; and (h) 400 nm shell. Representative TEM images of nanocatalysts with wide pore (i) 40 nm shell; (j) 60 nm shell; (k) 120 nm shell; and (l) 160 nm shell.

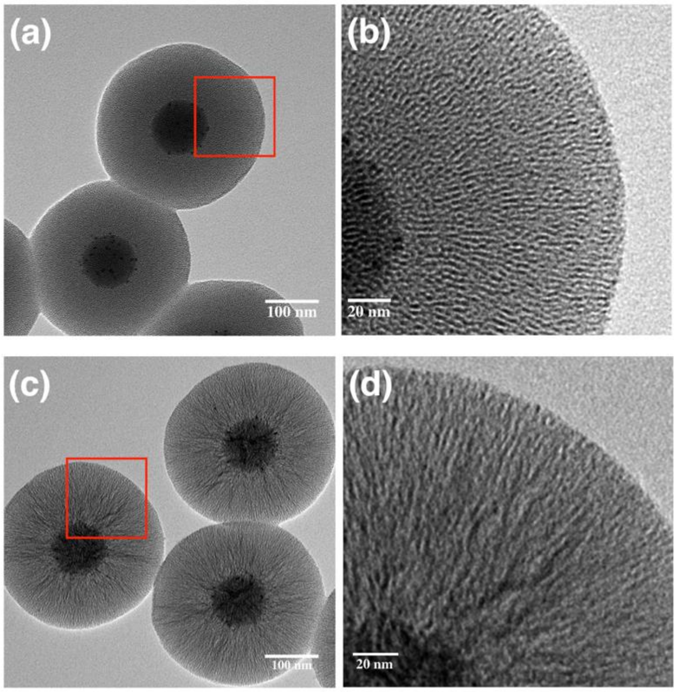

Supplementary Figure 2. TEM images of nanocatalysts. Representative TEM images of 120 nm shell nanocatalysts with (a,b) 2.2 and (c,d) 3.3 nm pore size. (b) and (d) are the magnified area (red rectangle) in respective (a) and (c) to show the pore structure.

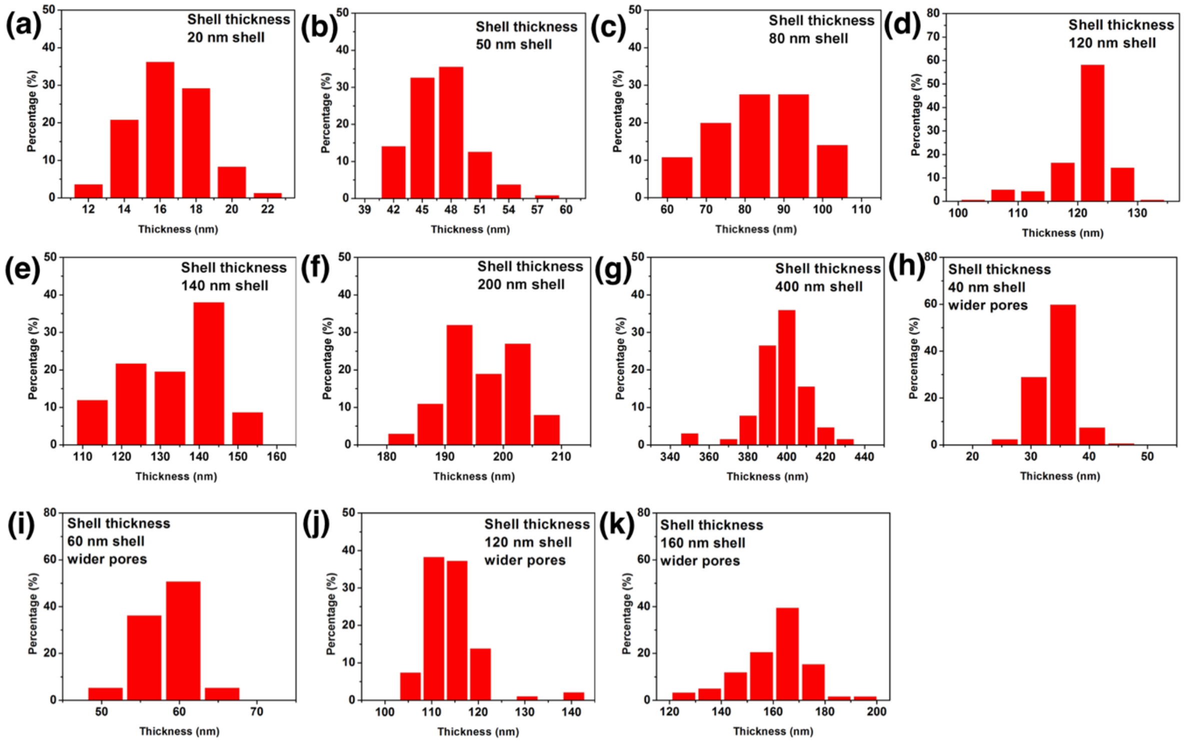

Supplementary Figure 3. Shell thickness distribution of nanocatalyst. The shell thickness value (Mean ± SD) of nanocatalysts with regular pore for (a) 20, (b) 50, (c) 80, (d) 120, (e) 140, (f) 200, (g) 400 shell was calculated as respective 16.5 ± 2.2, 48.5 ± 3.2, 83.1 ± 8.9, 118.5 ± 9.5, 132.8 ± 12.0, 199.1 ± 6.6, and 394.5 ± 14.1 nm respectively. The shell thickness value (Mean ± SD) of nanocatalysts with wider pore (3.3 nm) for (h) 40; (i) 60; (j) 120; and (k) 160 nm shell was calculated as 40.4 ± 3.2, 60.8 ± 3.4, 115.4 ± 5.8 and 162.8 ± 14.0 nm, respectively.

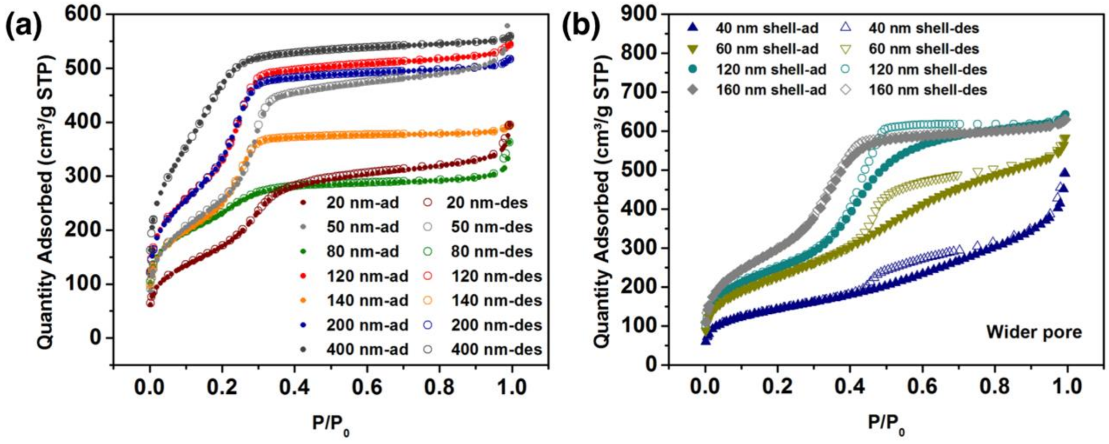

Supplementary Figure 4. Isotherm curves of (a) nanocatalysts with regular pore for 20, 50, 80, 120, 140, 200, and 400 nm shell. Isotherm curves of (b) nanocatalysts with wider pore for 40, 60, 120, and 160 nm shell.

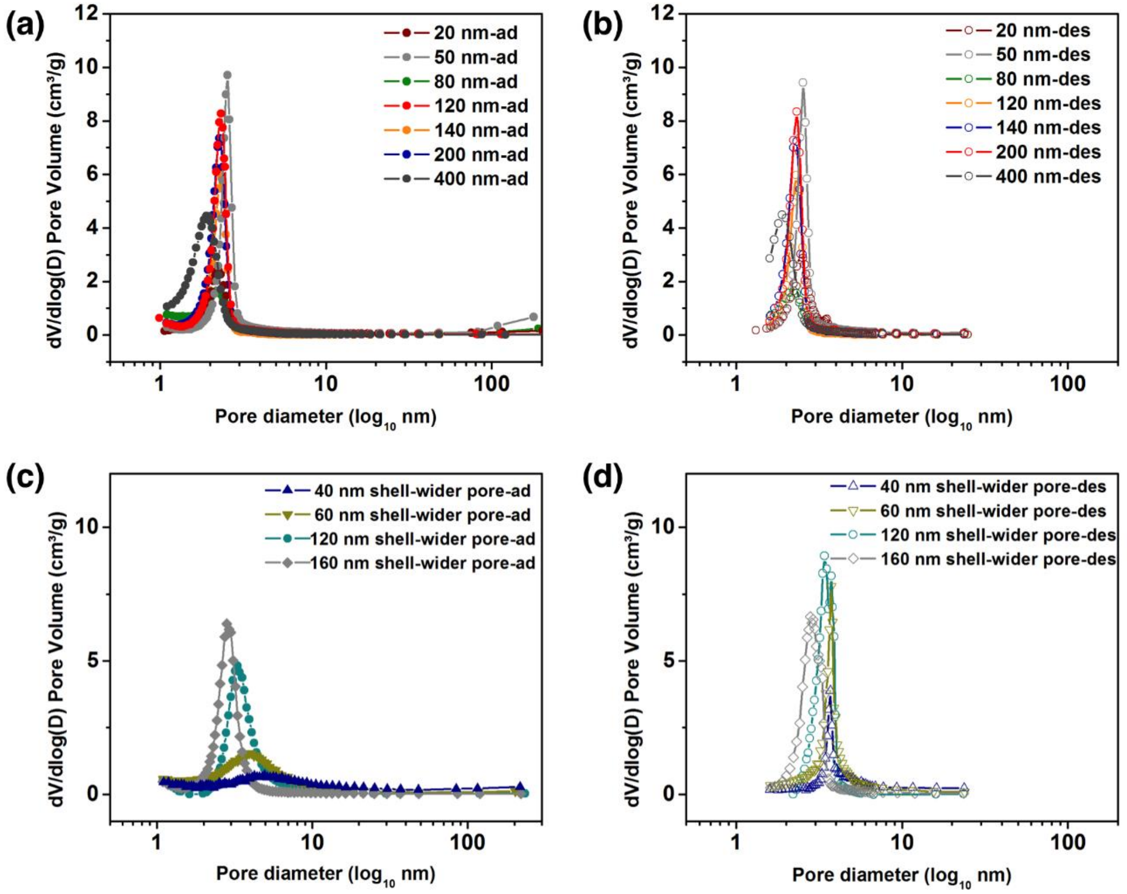

Supplementary Figure 5. Pore size distribution of nanocatalysts with regular pore for 20, 50, 80, 120, 140, 200, and 400 nm shell. (a) is the absorption branch and (b) is the desorption branch. The average pore size is around 2.2 nm. Pore size distribution of nanocatalysts with wider pore for 40, 60, 120, and 160 nm shell. (c) is the absorption branch and (d) is the desorption branch. The average pore size is around 3.0-3.5 nm.

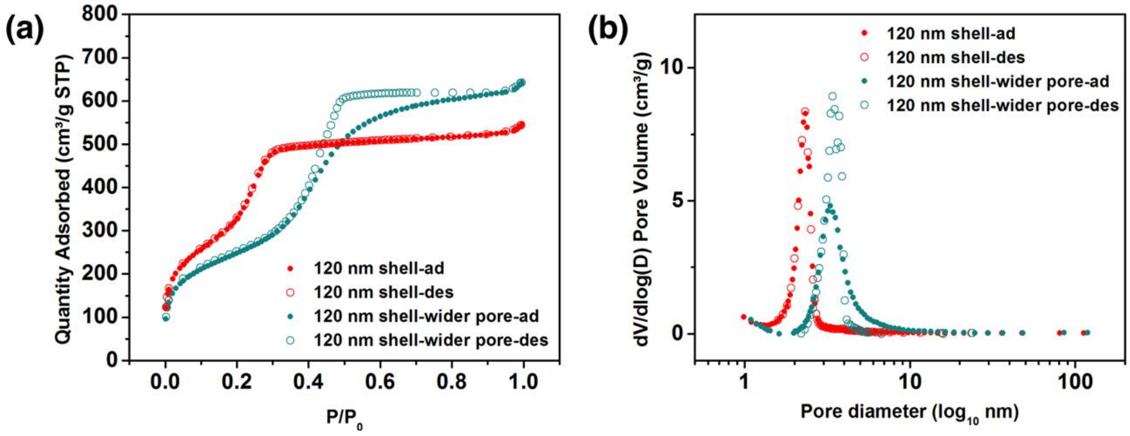

Supplementary Figure 6. Isotherm curves (a) and pore size distribution (b) of 120 nm shell nanocatalyst with regular 2.2 and wider 3.3 nm pore size.

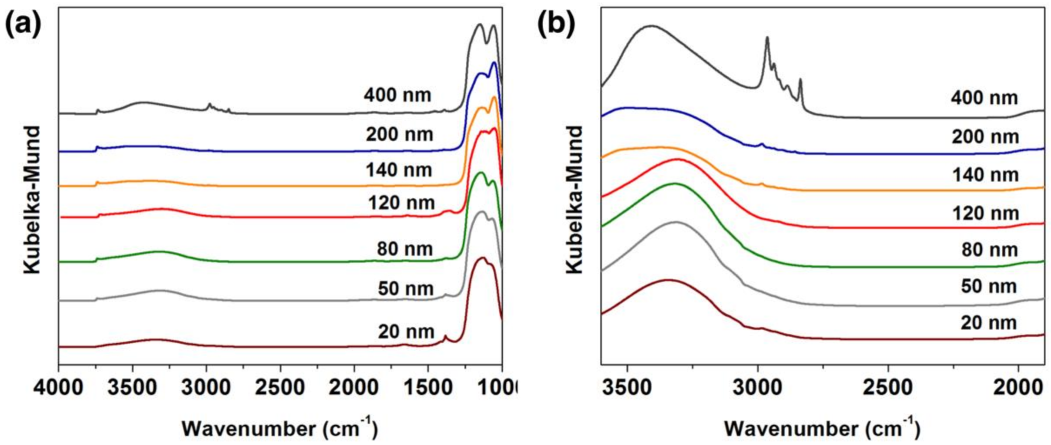

Supplementary Figure 7. Removing surfactant ligands in nanopores. (a) FTIR spectra of nanocatalysts with 20, 50, 80, 120, 140, 200, and 400 nm shell. (b) is the zoomed area of (a) in 1900-3600 cm^{-1}. All nanocatalysts have been diluted to prepare a 20 wt.% sample-KBr mixture, and treated at 120 °C for 30 min for dehydration. The nanocatalysts have slight C-H vibrations around 3000 cm^{-1}, which can be attributed to the remaining surfactants.

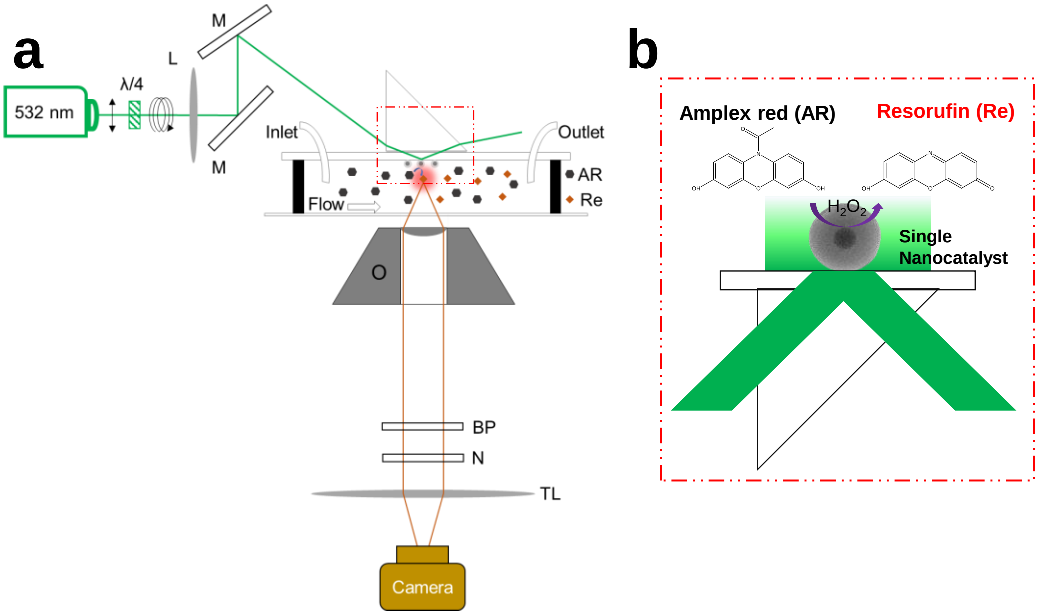

Supplementary Figure 8. Single molecule study of single nanocatalysts. (a) Prism-type total internal reflection fluorescence microscope with a flow cell for continuously supply of reactant. (b) Fluorogenic reaction where non-fluorescent amplex red is converted to highly fluorescent resorufin by 5 nm Pt NPs enraptured in the nanoporous shell of thickness of 120 nm.

Supplementary Note 1: single molecule imaging of catalytic reaction on single nanocatalysts

A prism-type total internal reflection fluorescence microscopy (TIRFM, Supplementary Fig. 8a) was built on a Nikon Ti-E inverted microscope for imaging generated resorufin molecules. A micro-flow chamber of 40 mm × 6 mm × 0.12 mm was assembled with the quartz slides and coverslip using double-side tape. A syringe pump was used to provide a continuous flow of constant and tunable concentrations of the reactant molecules in bulk solution and outside the nanopore at a rate of 20 μL min^{-1}.

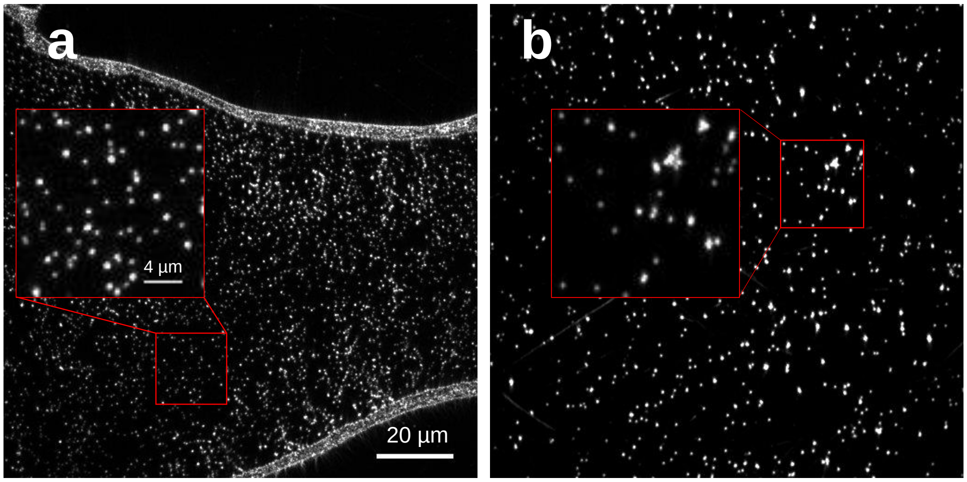

Supplementary Figure 9. TIRS images of 100 nm SiO_2@5 nm Pt@120 nm $mSiO_2$ nanocatalysts on quartz slides at high density (a) and low density (b). Zoom-in images show a better visualization of distributions of nanocatalysts on quartz slides.

Supplementary Note 2: density of particles on quartz slide surface

The density of 100 nm SiO_2@5 nm Pt@120 nm $mSiO_2$ nanocatalysts deposited on quartz slides was detected *in situ* with total internal reflection scattering (TIRS) microscopy (Supplementary Fig. 9)[7]. The TIRS imaging was performed on the same TIRF microscope but without the fluorescence filter set composing a 532 nm nortch filter and a 607/70 bandpass filter in the signal collecting path. An Andor iXonEM$^+$ 897 EMCCD camera was used to record the scattering images of the nanocatalysts. The TIRS images were taken immediately before recording the single particle catalytic events.

The drop-casting method for depositing nanocatalysts on quartz slides was tested and optimized. The images of individual spots in both supplementary figures indicated that the nanocatalysts were well separated and dispersed in methanol. For single particle imaging experiments, the density of nanocatalysts as in Supplementary Fig. 9b was chosen.

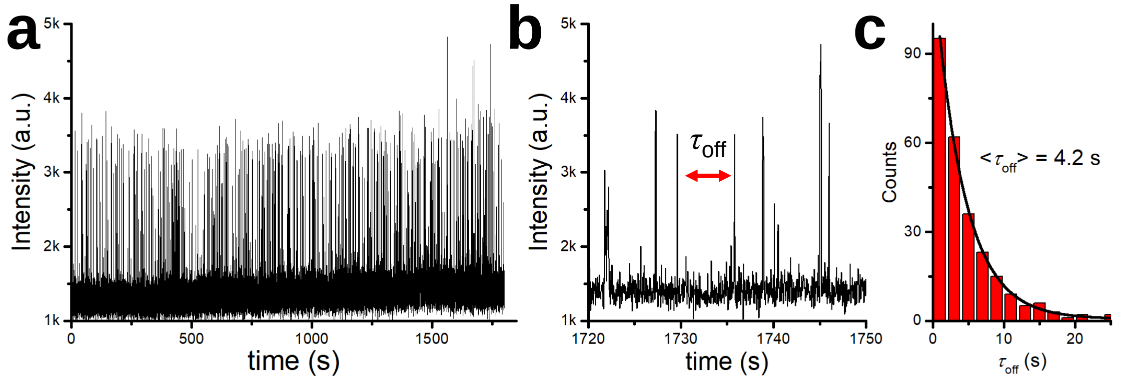

Supplementary Figure 10. Determine the catalytic reaction rate at single particle level with turnover resolution. (a) Typical fluorescence intensity trajectory from a single nanoparticle at a temporal resolution of 30 ms. (b) τ_{off} are defined as shown in the figure, which corresponds the interval time between two consecutive catalytic events. (c) Histogram distribution of τ_{off} over a long imaging period. Fitting the data points with single exponential decay function gives the average $< \tau_{off} > = 4.2 \pm 0.9$ s. Catalytic reaction rate can therefore be calculated to be $\upsilon_{rec} = < \tau_{off} >^{-1} = 0.24 \pm 0.05$ s^{-1}.

Supplementary Note 3: catalytic reaction kinetics at single particle level with turnover resolution

The distributions of τ_{off} (Supplementary Fig. 10c), which stands for the interval time between two consecutive catalytic events (Supplementary Fig. 10a, b), were calculated. Fitting the data points with exponential decay function[8] gave the mean delay time $<\tau_{off}>$ that was used as the characteristic value of waiting time for catalytic reactions. The inverse of the mean delay time $<\tau_{off}>^{-1}$ was used as the reaction rate υ_{rec}. This process was repeated for measuring the reaction rate over many single nanocatalysts and average reaction rates were determined under variable amplex red concentrations (Fig. 1b).

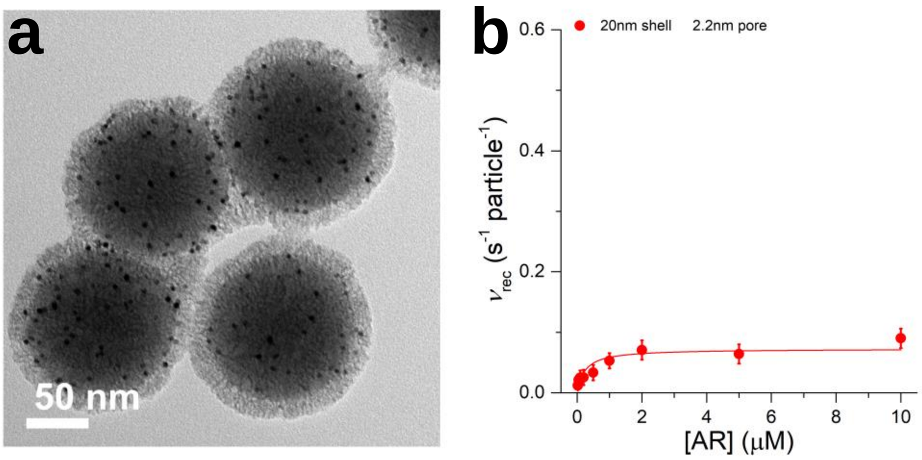

Supplementary Figure 11. Single particle reaction kinetics with 20 nm shell thickness. (a) High-resolution TEM images and (b) Reaction kinetics at single particle single molecule level. The error bars are calculated using catalytic reaction kinetic data from over 40 single nanocatalysts for each nanopore morphology.

Supplementary Note 4: catalytic reaction kinetics of 20 nm shell core-shell nanocatalysts

The adsorption/desorption equilibrium constant K_{AR} of 4.8 ± 0.9 μM^{-1} and rate constant k_{eff} of 0.072 ± 0.009 s^{-1} particle^{-1} were obtained for 20 nm shell thickness nanocatalysts from fitting the single particle single molecule kinetics data (Supplementary Fig. 1b). The smaller K_{AR} compared to that with no shell (K_{AR} = 6.1 ± 0.9 μM^{-1}) suggests the adsorption strength of AR is also reduced in 20 nm shell nanocatalyst due to the confinement effect. However, the confinement effect is weaker compared to that in thicker shells (Fig. 1c). On the other hand, the k_{eff} is only slightly larger for 20 nm shell nanocatalyst than that with no shell (k_{eff} = 0.068 ± 0.007s^{-1} particle^{-1}). This is because of the defects and non-uniformity of the shell structure visible in TEM images (Supplementary Fig. 11a), which leads to an intermediate confinement effects between nanocatalyst with no shell and those with thicker shells.

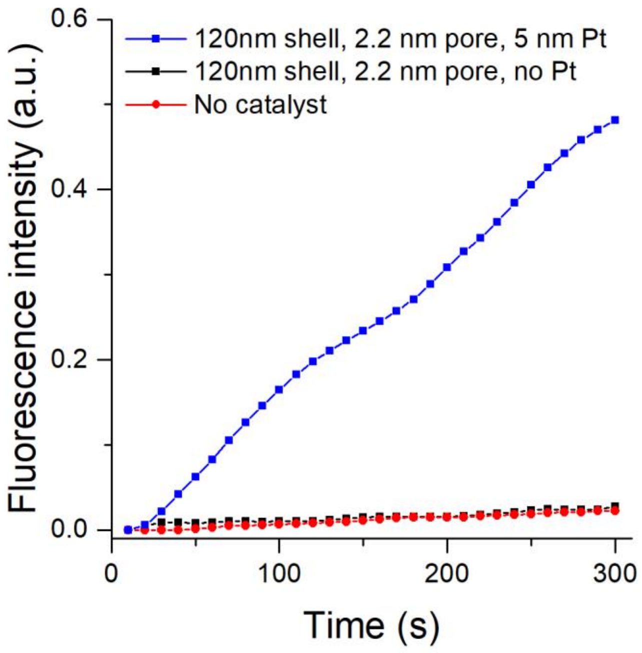

Supplementary Figure 12. Measure catalytic reaction rates with/without nanocatalysts at ensemble level. Concentration of 120 nm shell, 2.2 nm pore nanocatalyst at ~ 10^{11} particles mL^{-1} were used in the experiments. A reaction solution of 2 µM amplex red, 50 mM hydrogen dioxide in 10mM PBS buffer (pH 7.4) was used for the experiments.

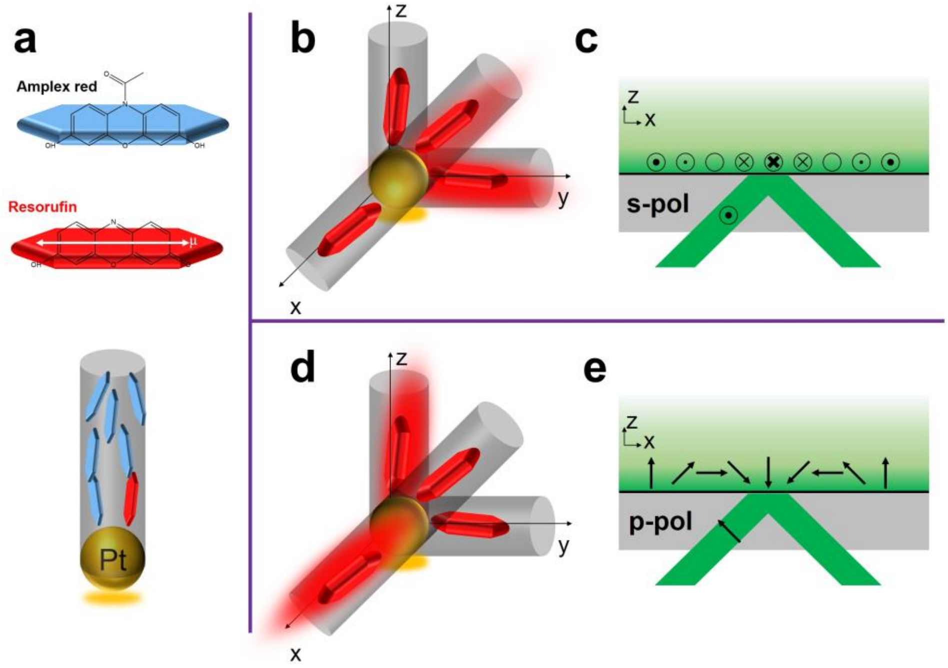

Supplementary Figure 13. Study molecular orientations in nanopore using single molecule fluorescence polarisation microscopy.

Supplementary Note 5: single molecule fluorescence polarisation microscopy

The molecular sizes (1.3 nm × 0.7 nm) of amplex red (AR) and resorufin (Re) are comparable to the nanopore size (2.2 nm and 3.3 nm diameter); therefore, motions (especially rotations) of these molecules would be strongly restricted in the nanopores. It should be noted that because AR and Re have similar molecular structures, the experimental results from imaging the fluorescent product Re, which has a dipole moment along the long axis of the molecule (Supplementary Fig. 13a), were applied to understand the motions of the non-fluorescent reactant AR.

Single molecule fluorescence polarisation microscopy imaging was used to verify the restricted motions of Re molecules inside the nanopore. Fluorescent molecules show the highest brightness when their absorption transition dipole moments are parallel to the polarisation direction of the excitation light. Two linear polarisation directions, s-pol and p-pol can be realized in TIRFM[9]. The

s-pol direction is perpendicular to the light propagation direction and parallel to the interface (Supplementary Fig. 13c), while the p-pol direction remains in the same plane of incident light, containing both the transverse and longitudinal components (Supplementary Fig. 13e). The restricted molecular orientations of Re molecules in different sets of nanopores will be efficiently excited by the linearly polarised light (Supplementary Fig. 13b, d). When a circularly polarised (c-pol) light is used for excitation, all Re molecules in nanopores with different absorption dipole moment directions should be excited equally in the evanescent field. In our molecular orientation measurement experiments (Fig. 2), both s-pol and c-pol incident light were used for the imaging study.

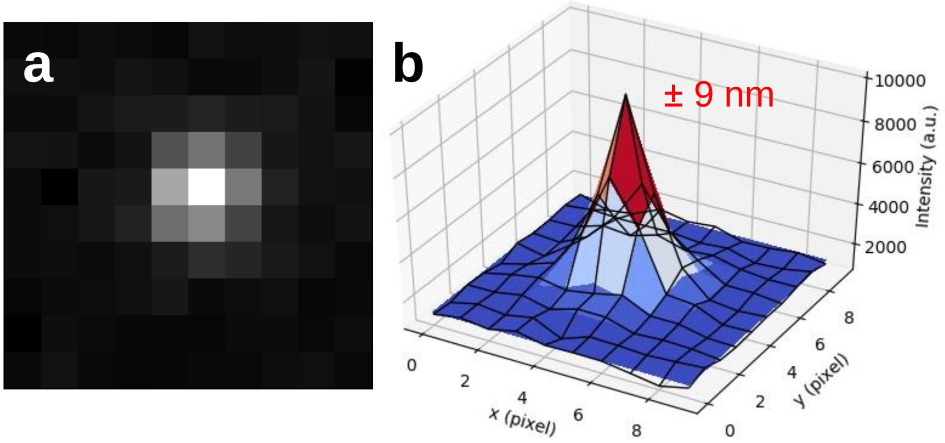

Supplementary Figure 14. Localise single molecular positions with nanometre precision. (a) Typical single frame image of fluorescent products on nanocatalysts. (b) The centre position of fluorescent bursts can be determined with a precision of a few nanometres by 2D Gaussian fitting of the fluorescence profile. In this example, the centre position is determined with a precision of 9 nm. The localisation precision varies with the fluorescence signal collected from each resorufin molecule.

Supplementary Note 6: localise single-molecule catalytic events with nanometre precision

Supplementary Fig. 14a is an example of a fluorescence image of resorufin molecule in nanopore. The positions of identified molecules were localised by using a similar approach published previously [8]. The localisation precision largely depends on both the photon number collected from the individual fluorescent molecule and the fluorescence background noise. The following equation is typically used to evaluate localisation precision [10],

$$\sigma = \sqrt{\frac{s^2}{N} + \frac{a^2}{12N} + \frac{8\pi b^2 s^4}{a^2 N^2}} \quad \text{.......}(1)$$

where s is the standard deviation of the Gaussian distribution that equals 1/2.2 of the PSF width, a is the pixel size, b is background noise, and N is the number of collected photons. The three components that affect the molecular position uncertainties can be attributed to the photon noise (s^2/N), the effect of the finite pixel size (a) of the detector, and the effect of the background noise

(*b*) respectively. For the example shown in Supplementary Fig. 14b, a localisation precision of 9 nm was determined. The nanometre precision of molecular positions enabled us to localise the generation of single resorufin molecules through the catalytic reaction and track their mass transport inside nanopores. Nonetheless, the localisation precision also varies regarding the spatial location of the resorufin molecules during the whole catalytic reaction processes. Resorufin molecules located near the quartz slide surface versus those are far away can be determined with higher localisation precision since more photons can be collected by the objective. To quantitative analysing the large scale of imaging data, a self-written MATLAB script was used to identify catalytic events, localize molecule positions, and following up a statistical analysis of reaction kinetics.

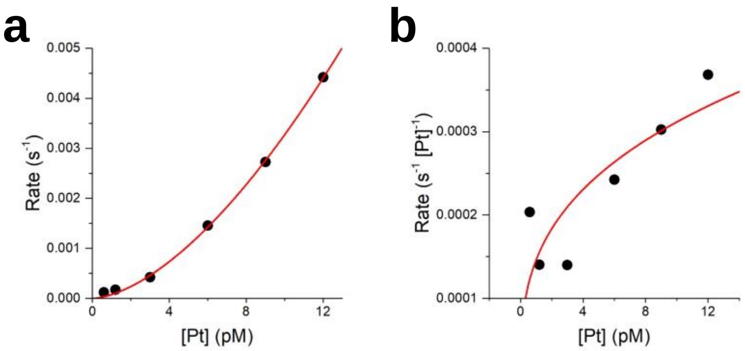

Supplementary Figure 15. Ensemble experiments measuring the dependence of activities on concentrations of nanocatalysts. (a) Initial reaction rates under low concentrations (0.6-12 pM) of nanocatalysts without nanoporous shell (100 nm SiO_2@5 nm Pt). (b) Normalized Initial reaction rates (over nanocatalyst concentrations) at different concentrations of nanocatalysts.

Supplementary Note 7: molecular mechanism of the oxidation reaction of amplex red to resorufin

Catalytic reaction rates under low concentrations (0.6-12 pM) of nanocatalysts without nanoporous shell (100 nm SiO_2@5 nm Pt) were measured using ensemble experiments (Supplementary Fig. 15a). The measured catalytic reaction rates (i.e. the initial rate of resorufin formation) over nanocatalyst concentrations did not follow a linear or first-order relationship as would be expected if resorufin were formed directly from amplex red. Fitting the data points, a nonlinear relationship with a fractional order of 1.6 was determined thus the catalytic reaction is more close to a second-order kinetic mechanism where indicating the likely reaction between two amplex red radical molecules. Moreover, we also normalized the reaction rates over the nanocatalyst concentration. As shown in Supplementary Fig. 15b, the normalized reaction rates increase as the concentration of nanocatalyst gets higher as well, in agreement with previously reported results [11]. If the reaction mechanism follows a first-order reaction mechanism, the normalized reaction rates should have

remained to be similar. Therefore the mechanism of oxidation of amplex red to resorufin on platinum nanoparticles should also follow successive steps of single-electron transfer processes [12,13] as shown in Supplementary Fig. 16. In the initial step of the reaction, phenol group on AR transfers one electron to reactive oxygen species (ROS) e.g., chemisorbed oxygen on Pt surface[14] to produce the intermediate species of AR radical ($AR^{\bullet}$). The following disproportionation reaction requires the participation of two $AR^{\bullet}$ to form one AR molecule and AR^{+} cation. AR^{+} can be further converted to the final product Re through hydrolysis.

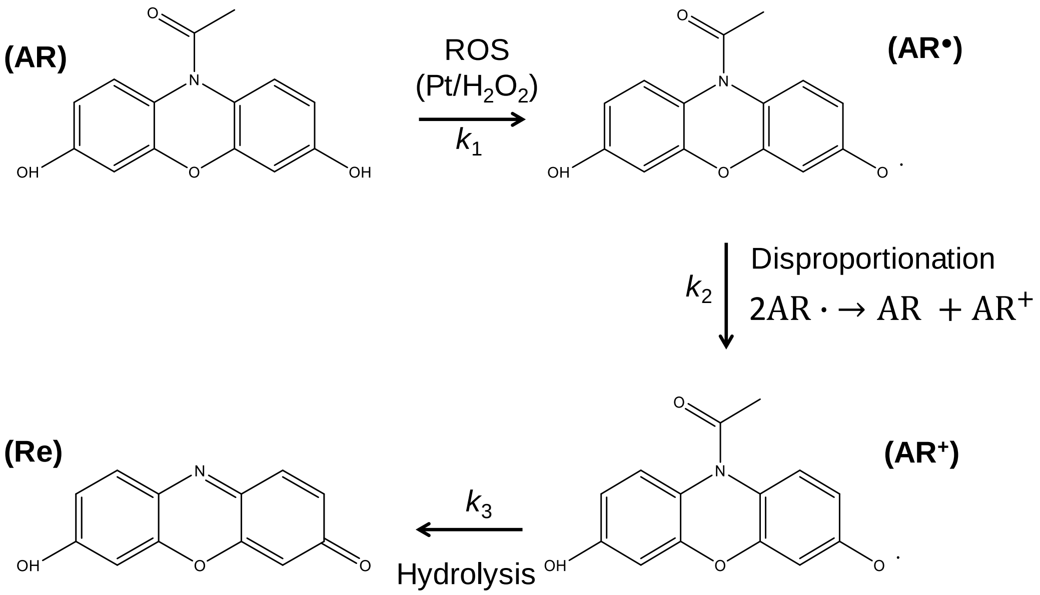

Supplementary Figure 16. Reaction mechanism of the oxidation of amplex red to resorufin.

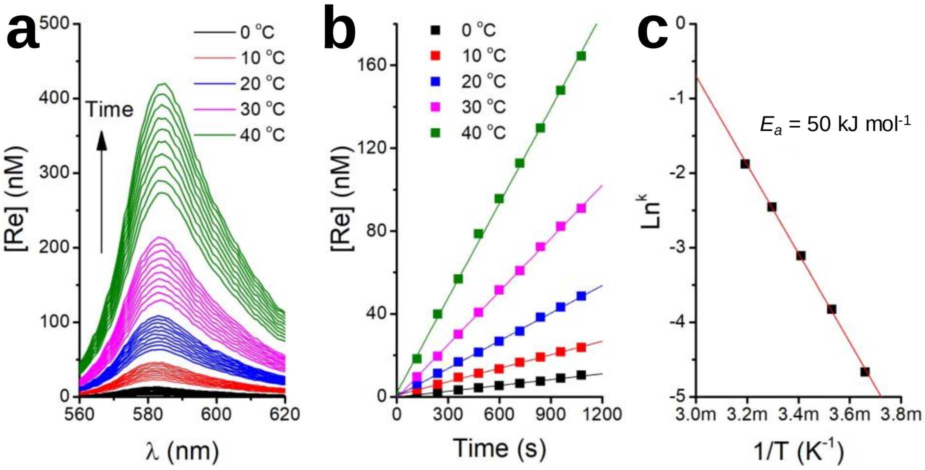

Supplementary Figure 17. Ensemble measurement of reaction activities of nanocatalysts with 120 nm porous shell, 2.2 nm pore under different temperature. (a) Concentrations of product molecule resorufin increase over 20 minutes for each controlled temperatures from 0 ^{o}C to 40 ^{o}C. (b) Determine the reaction activities of nanocatalysts using the formation rate of resorufin by linearly fitting the data points. (c) Activation energy of E_a = 50 kJ mol^{-1} was measured by fitting the reaction rates over the inverse of absolute temperature according Arrhenius equation.

Supplementary Note 8: chemical reaction activation energy measurement

To further verify the increased chemical conversion rate of amplex red on platinum nanoparticles in nanopores, the activation energy of reaction was measured (Supplementary Methods 4, Supplementary Fig. 17). The clearly lower activation energy was observed for nanocatalysts with nanoporous shell than that without nanoporous shell (Supplementary Fig. 18). Moreover, the activation energy of oxidation of amplex red is similar with different nanopore length but different when the nanopore diameter changes. The results here as well as the measurement of molecular adsorption suggest that the nanoconfinement effects can potentially change the catalytic activity through tuning the adsorption strength of molecules.

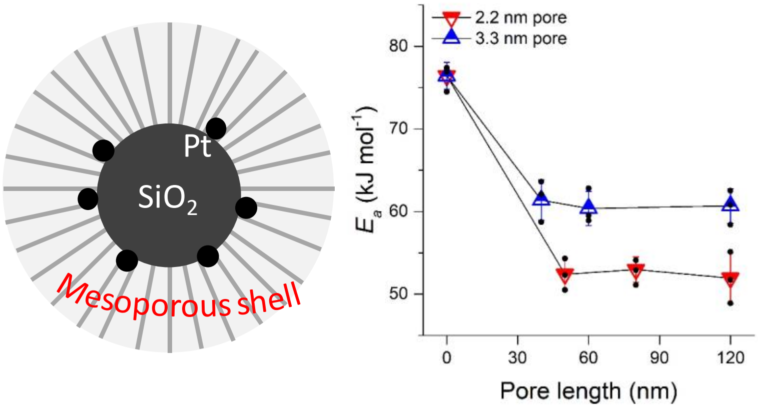

Supplementary Figure 18. Nanoconfinement effect on activation energy of the oxidation reaction of amplex red with variable pore length and pore diameter.

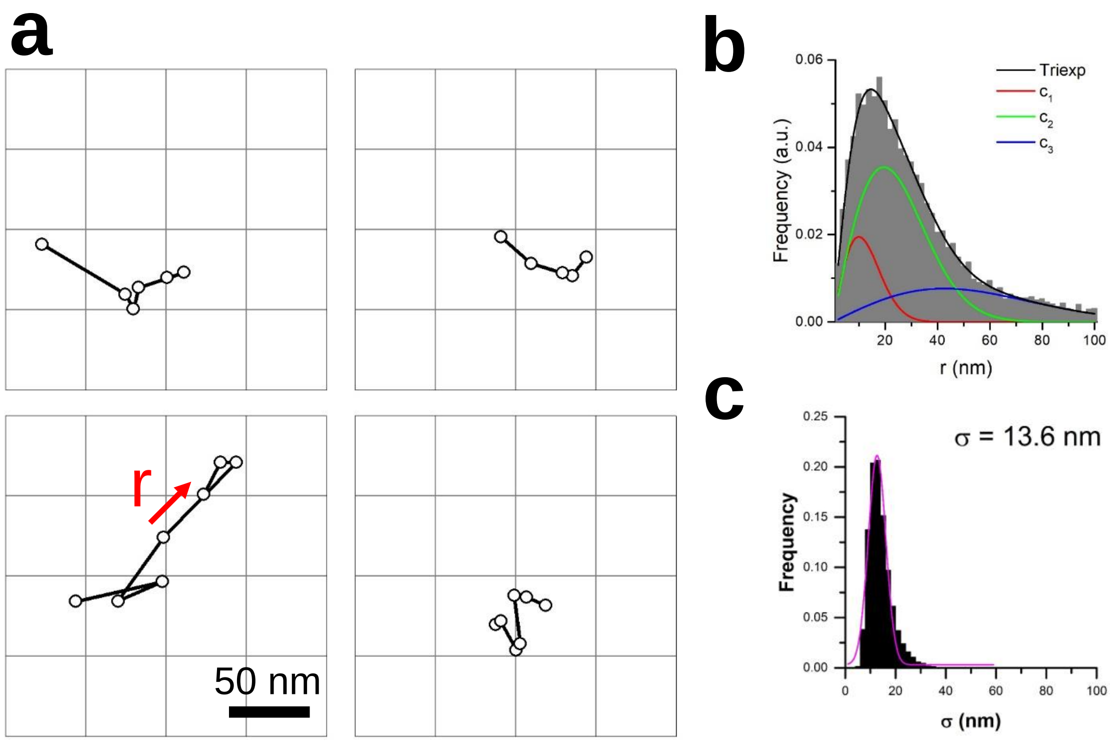

Supplementary Figure 19. Global analysis of the diffusion of resorufin inside 120 nm nanopores. (a) Typical single molecular trajectories. (b) MSD analysis of jump distances between consecutive frames of trajectories of resorufin molecule in nanopores and (c) uncertainty in localising molecular positions.

Supplementary Note 9: mass transport of resorufin inside nanopore

Supplementary Fig. 19a shows four representative trajectories of resorufin molecules inside the nanopores. Mean squared displacement (MSD) analysis was used to analysing single molecular trajectories. The global analysis strategy, in which large datasets of single molecular trajectories are used to draw conclusions with statistical significance, was used for achieving higher accuracy because of the relatively short lifetime of resorufin molecule inside nanopore. Supplementary Fig. 19b and c show the square root of displacement (*r*) in resorufin molecular trajectories and the

localisation uncertainty (σ). The much larger r when comparing to σ suggests that resorufin is undergoing diffusive transport rather than permanently adsorbed at certain sites inside nanopore.

To quantitatively measure the molecular transport of resorufin inside nanopore, we fitted the distribution of molecular displacement with the probability density function [15]:

$$p(r^2, t) \cdot dr^2 = \frac{1}{\pi\langle r^2_{(t)}\rangle} \exp\left(\frac{-r^2_{(t)}}{\langle r^2_{(t)}\rangle + \sigma^2}\right) 2\pi r \cdot dr^2 \qquad (2)$$

where $\langle r^2\rangle$ stands for the mean square displacement and σ is the localisation uncertainty. In heterogeneous environments, the molecular transport can be described by a multicomponent probability density function with distinct diffusion coefficients.

$$p(r^2, t) \cdot dr^2 = \sum_1^i c_i \frac{1}{\pi\langle r^2_{i(t)}\rangle} \exp\left(\frac{-r^2_{(t)}}{\langle r^2_{i(t)}\rangle + \sigma^2}\right) 2\pi r \cdot dr^2 \qquad (3)$$

The diffusion coefficient was determined using equation (4) based on the Einstein-Smoluchowski equation for random diffusion in one dimension [16,17].

$$\langle r^2_{i(t)}\rangle = 2D_i \cdot t \qquad (4)$$

To best interpret the MSD distributions, it requires to use radial probability density function with three components (denoted as c_1, c_2, c_3, Supplementary Fig. 19b) for fitting the data points resulting three distinct characteristic diffusion coefficients (D_1, D_2, D_3). The distinct diffusion coefficients indicate that the local environments inside nanopore are heterogeneous. Therefore, the transport of resorufin molecules in nanopores should be a combination of different motion modes, including surface adsorption and inner pore diffusion, instead of just a simple Brownian motion. The medium diffusion rate (c_2), which contains significant contributions from both c_1 (adsorption) and c_3 (random movement), dominates the molecular transport.

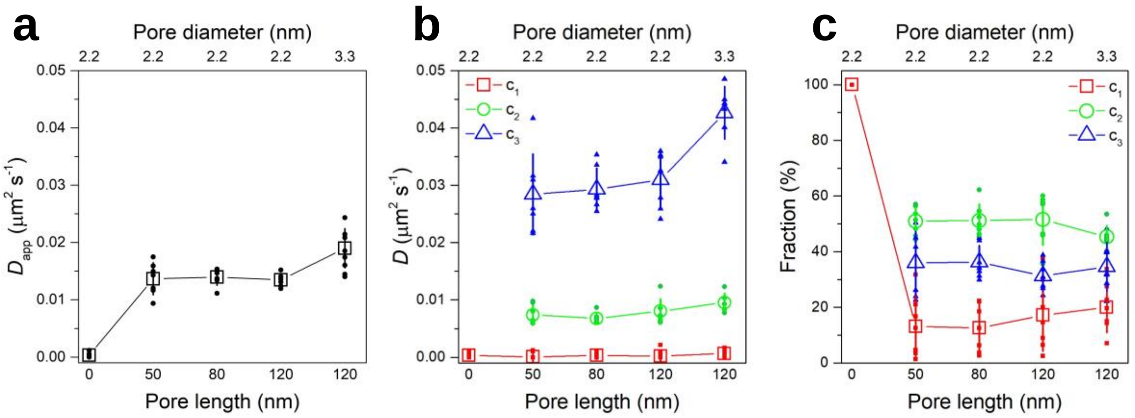

Supplementary Figure 20. MSD analysis of molecular transport in different nanopore morphologies. (a) Measured apparent diffusion coefficients of resorufin transporting in the nanopores of different length and diameter. Diffusion coefficients (b) and corresponding fractions (c) of the three sub-populations (c_1, c_2, c_3) when using multicomponent probability density function to best fit the displacement distribution data. Error bar are calculated from MSD analysis of single molecular trajectories at all amplex red concentrations.

Supplementary Note 10: the effects of nanopore morphology on mass transport

The molecular diffusion inside nanoconfined space is complicated and determined by many factors such as substrate-surface interactions, substrate-substrate interactions, and substrate-solvent interactions.[18] Moreover, the geometry of the local environment also plays an important role in controlling the molecular movement. Here, we had investigated the roles of nanopore length and diameter in changing the mass transport behaviors of resorufin molecules.

As shown in Supplementary Fig. 20a, the apparent diffusion coefficients under different porous shell thickness show small variations. This also holds true for the diffusion coefficients of the three sub-populations of molecular motions (Supplementary Fig. 20b). The fractions of sub-populations under different porous shell thicknesses were also similar (Supplementary Fig. 20c). However, molecular transport is sensitive to the diameter of the nanopore. The apparent diffusion coefficient

of 3.3 nm nanopore is larger than the case of 2.2 nm nanopore even though their pore lengths are both 120 nm. The increased apparent diffusion coefficients for the larger nanopore was mostly caused by the faster diffusion portion (c_3, Supplementary Fig. 20b) even though its fraction was slightly decreased (c_3, Supplementary Fig. 20c). The single molecular trajectory analysis provides accurate measurement of molecular diffusion in nanopores under reaction conditions, which then allows us to carry out further analysis to decouple the influences of molecular transport and reaction kinetics [19].

Supplementary References

1 Hartlen, K. D., Athanasopoulos, A. P. T. & Kitaev, V. Facile Preparation of Highly Monodisperse Small Silica Spheres (15 to >200 nm) Suitable for Colloidal Templating and Formation of Ordered Arrays. *Langmuir* **24**, 1714-1720 (2008).

2 Zhang, Y. *et al.* Highly Selective Synthesis of Catalytically Active Monodisperse Rhodium Nanocubes. *J. Am. Chem. Soc.* **130**, 5868-5869 (2008).

3 Pei, Y. C. *et al.* Tuning surface properties of amino-functionalized silica for metal nanoparticle loading: The vital role of an annealing process. *Surf. Sci.* **648**, 299-306 (2016).

4 Han, R. *et al.* Geometry-assisted three-dimensional superlocalization imaging of single-molecule catalysis on modular multilayer nanocatalysts. *Angew. Chem. Int. Ed.* **53**, 12865-12869 (2014).

5 Xiao, C. *et al.* High-temperature-stable and regenerable catalysts: platinum nanoparticles in aligned mesoporous silica wells. *ChemSusChem* **6**, 1915-1922 (2013).

6 Yue, Q. *et al.* An Interface Coassembly in Biliquid Phase: Toward Core-Shell Magnetic Mesoporous Silica Microspheres with Tunable Pore Size. *J. Am. Chem. Soc.* **137**, 13282-13289 (2015).

7 Marchuk, K., Ha, J. W. & Fang, N. Three-Dimensional High-Resolution Rotational Tracking with Superlocalization Reveals Conformations of Surface-Bound Anisotropic Nanoparticles. *Nano Lett.* **13**, 1245-1250 (2013).

8 Ha, J. W. *et al.* Super-Resolution Mapping of Photogenerated Electron and Hole Separation in Single Metal–Semiconductor Nanocatalysts. *J. Am. Chem. Soc.* **136**, 1398-1408 (2014).

9 Axelord, D. *Chapter 7: Total Internal Reflection Fluorescence Microscopy*. (2008).

10 Thompson, R. E., Larson, D. R. & Webb, W. W. Precise Nanometer Localization Analysis for Individual Fluorescent Probes. *Biophys. J.* **82**, 2775-2783 (2002).

11 Han, K. S., Liu, G., Zhou, X., Medina, R. E. & Chen, P. How Does a Single Pt Nanocatalyst Behave in Two Different Reactions? A Single-Molecule Study. *Nano Lett.* **12**, 1253-1259 (2012).

12 Dębski, D. *et al.* Mechanism of oxidative conversion of Amplex® Red to resorufin: Pulse radiolysis and enzymatic studies. *Free Radic. Biol. Med.* **95**, 323-332 (2016).

13 Gorris, H. H. & Walt, D. R. Mechanistic Aspects of Horseradish Peroxidase Elucidated through Single-Molecule Studies. *J. Am. Chem. Soc.* **131**, 6277-6282 (2009).

14 Serra-Maia, R. *et al.* Mechanism and Kinetics of Hydrogen Peroxide Decomposition on Platinum Nanocatalysts. *ACS Appl. Mater. Interfaces* **10**, 21224-21234 (2018).

15 Barlow, R. J. Statistics, A Guide to the Use of Statistical Methods in the Physical Sciences. *John Wiley & Sons: Chichester* (1989).

16 Roeffaers, M. B. J. *et al.* Spatially resolved observation of crystal-face-dependent catalysis by single turnover counting. *Nature* **439**, 572-575 (2006).

17 Berg, H. C. Random Walks in Biology. *Princeton University Press: Princeton* (1993).

18 Higgins, D. A., Park, S. C., Tran-Ba, K.-H. & Ito, T. Single-Molecule Investigations of Morphology and Mass Transport Dynamics in Nanostructured Materials. *Annu. Rev. Anal. Chem.* **8**, 193-216 (2015).

19 Dong, B. *et al.* In situ quantitative single-molecule study of dynamic catalytic processes in nanoconfinement. *Nat. Catal.* **1**, 135-140 (2018).
